# Visual statistical learning requires attention

**DOI:** 10.3758/s13423-024-02605-1

**Published:** 2024-11-04

**Authors:** Dock H. Duncan, Dirk van Moorselaar, Jan Theeuwes

**Affiliations:** 1 Department of Experimental and Applied Psychology, Vrije Universiteit Amsterdam, Amsterdam, the Netherlands; 2 Institute Brain and Behavior Amsterdam (iBBA), Amsterdam, the Netherlands; 3 William James Center for Research, ISPA-Instituto Universitario, Lisbon, Portugal

**Keywords:** Statistical learning, Attention, Visual search, Distractor suppression, Feature guided search

## Abstract

Statistical learning is a person’s ability to automatically learn environmental regularities through passive exposure. Since the earliest studies of statistical learning in infants, it has been debated exactly how “passive” this learning can be (i.e., whether attention is needed for learning to occur). In Experiment 1 of the current study, participants performed a serial feature search task where they searched for a target shape among heterogenous nontarget shapes. Unbeknownst to the participants, one of these nontarget shapes was presented much more often in location. Even though the regularity concerned a nonsalient, nontarget item that did not receive any attentional priority during search, participants still learned its regularity (responding faster when it was presented at this high-probability location). While this may suggest that not much, if any, attention is needed for learning to occur, follow-up experiments showed that if an attentional strategy (i.e., color subset search or exogenous cueing) effectively prevents attention from being directed to this critical regularity, incidental learning is no longer observed. We conclude that some degree of attention to a regularity is needed for visual statistical learning to occur.

## Introduction

Our past experiences guide future behavior. In the case of attention, previous selection experiences allow the extraction and learning of regularities present in a display; a process often referred to as visual statistical learning (VSL). Statistical learning is thought to be a fundamental cognitive mechanism underlying human procedural learning (Frost et al., 2019; Turk-Browne, 2012); and in the case of attention, VSL enables observers to extract the distributional properties from sensory input across space and time, making it possible to adapt attentional selection priorities to the regularities present in the environment (Frost et al., 2015; Theeuwes et al., 2022). VSL can be labelled as a “selection history” effect, which makes up the modern conceptualization of the attention system along with the observer’s top-down goals and the current scene’s bottom-up saliency characteristics (Anderson et al., 2021; Awh et al., 2012; Failing & Theeuwes, 2018; Theeuwes, 2018, 2019).

Paradigms such as contextual cuing (whereby search for a target is more efficient when displays have been previously searched; Chun, 2000; Chun & Jiang, 1998, 2003; Goujon et al., 2015), probability cuing (where search is much faster when targets appear more often in high-probability locations in space; Duncan, Theeuwes, et al., 2023a; Ferrante et al., 2018; Geng & Behrmann, 2005; Huang et al., 2022; Jiang et al., 2013), and object-based learning (Jeong & Cho, 2024; van Moorselaar & Theeuwes, 2023, 2024) have demonstrated how the visual system leverages its experience to encode environmental regularities and improve visual search. These paradigms demonstrate that participants implicitly encode the statistical properties of their search target, and provide the basis for the once common stance that VSL only occurs for task relevant items (Turk-Browne, 2012; Turk-Browne et al., 2005; Vadillo et al., 2020).

However, recent results have demonstrated that learning can also occur for task-irrelevant distractors that capture attention in an exogenous (bottom-up) way (Failing, Wang, et al., 2019b; Gao & Theeuwes, 2020; Goschy et al., 2014; van Moorselaar & Theeuwes, 2022; Wang & Theeuwes, 2018a). This learning is expressed via learned suppression of this distracting input and runs counter to previous claims that learning can only occur for stimuli that are task relevant, demonstrating instead that VSL is more ubiquitous than previously thought (see Theeuwes et al., 2022, for a review). Furthermore, VSL has also been shown to persist (though hindered in some cases) despite high working memory load (Gao & Theeuwes, 2020; Giménez-Fernández et al., 2023; Manginelli et al., 2013; Vickery et al., 2010; Won & Jiang, 2015; but see Amsalem et al., 2023), a process known to interfere with attentional processing (Awh & Jonides, 2001; Cowan et al., 2005; Theeuwes et al., 2009). Such results lay the foundation upon which some claim that VSL is an entirely attention-independent mechanism (Hansmann-Roth et al., 2021; Jiang & Leung, 2005).

When discussing the role of attention in VSL, one should consider that attention control has traditionally been characterized as resulting from the dynamic interplay between the physical properties of the object (bottom-up attention) and the goals of the observer (top-down attention). Importantly, if a solitary item is presented on a uniform background then bottom-up attention, the visual systems attraction to salient events, will automatically be directed towards this item. Consequently, shape or sequence learning experiments where single items are successively presented on the screen are inherently ill-suited to investigate whether learning requires attention. Instead, to rule out a role of bottom-up attention, the experimental design needs to incorporate multi-item arrays such that the object that contains the regularity no longer pops out in the scene. By contrast, goal-directed top-down attention describes how the attentional system selects task-relevant features and orients attention towards potential targets. While it has been convincingly demonstrated that VSL does not require top-down attention to occur (Duncan & Theeuwes, 2020; Turatto et al., 2018), these experiments did not set out to also control the influence of bottom-up attention, leaving open the possibility that observed learning was driven by exogenous capture and precluding the conclusion that VSL can occur in the total absence of attention. Furthermore, several studies have shown that VSL effects scale with the degree of top-down attention directed to the regularities (Duncan & Theeuwes, 2020; Jiang & Chun, 2001; Musz et al., 2015), necessitating experimental designs that are sensitive to small effect sizes if these effects are to be studied adequately (Brysbaert, 2019).

**Fig. 1:**
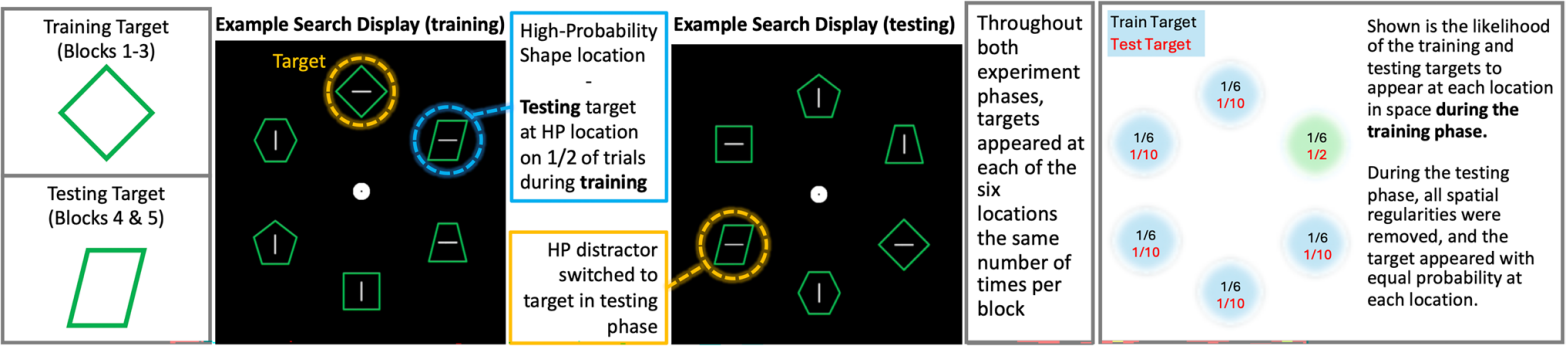
Example training and test displays with high-probability location indicated. (Left) Example training and test target shape identities are examples only; target identities assigned randomly at the beginning of the experiment for each participant. Participants’ task were to find this shape target on every trial and report whether the grey line within this shape was oriented horizontally or vertically. After three blocks (training), participants’ search target was changed to a different shape and were given the exact same task for an additional two blocks (testing). The shape that would be the search target during the testing display was presented more frequently at one location during training (its high-probability location) but equally frequently at all locations during testing. Note also that colored circles were not present in the actual experiment; participants only saw green shapes on a black background. (Right) Visualization of the spatial distribution of training and testing targets during the training phase of the experiment. Note that while the training target appeared at each of the six locations the same number of times, the testing target had an imbalanced spatial distribution. During the test phase, the test target appeared with equal likelihood at each of the six possible locations, thus mirroring the spatial distribution of the training target during the training phase. (Color figure online)

To investigate the influence of attention on VSL, special care must be taken to design an experiment that controls both the top-down and bottom-up attention attraction to an embedded regularity in a task. To achieve this, we used a visual search task (Fig. 1) in which observers had to search for a specific target shape (e.g., a square) among heterogeneous nontarget shapes (e.g., diamonds, hexagons), a task design that mitigates the influence of bottom-up attention by making all search items equally salient (Egeth et al., 1984; Leber & Egeth, 2006). Crucially, rather than having a regularity related to the search target or a singleton distractor, as has previously been done in visual search studies (Jiang et al., 2013; Wang & Theeuwes, 2018a), one of the nontarget, nonsalient shapes appeared instead more often in one location during training. This shape should have received very little, if any, attentional priority, making it a prime candidate to study the role of attention and statistical learning. Following an initial training phase, in which this spatial regularity was present, during a subsequent testing phase, all shapes appeared in each location with equal likelihood and participants now had to search for the shape that previously had a high-probability location. It has been demonstrated previously that VSL effects persist for a period before extinction (Duncan & Theeuwes, 2020; Jiang et al., 2013; Sauter et al., 2019), meaning that if learning had occurred in the training phase, an effect of shape location should also be observed in this testing phase when the target shape appeared at its previously high-probability location. This design allowed us to examine whether observers can learn regularities regarding nonsalient nontarget shapes or alternatively, as suggested by some (Anderson et al., 2021; Theeuwes et al., 2022), that such learning is restricted to singletons that are salient enough to summon attention on every trial.

To foreshadow the results, in Experiment 1 it was found that observers can also learn regularities regarding nonsalient, nontarget shapes, providing another striking example of the visual system’s sensitivity to regularities within the environment. Yet while the experimental design precluded a role of bottom-up attention, it is premature to conclude that VSL can occur in the absence of attention based on these findings as alternative explanations, such as serial search transiently directing attention towards this regularity, or nonzero attentional allocation towards this regularity due to shared color features, prohibits this conclusion. In a pair of follow-up experiments using color subset search (Experiment 2) and exogenous cueing (Experiment 3), we show that when attentional strategies successfully prevent attention from being directed towards shape regularity during training, incidental learning ceases to occur. This highlights the essential role that attention plays in statistical learning.

## Experiment 1

### Method

#### Participants

Following an extensive pilot experiment, main effects of nontarget shape location were expected to be roughly *d* = 0.29 in training and testing phases. Given this target, we chose a sample size of 120 participants for this experiment, which represented an 85% confidence for detecting effects as low as *d* = 0.29 (preregistrations & pilot study: https://osf.io/q3ubp).

One hundred and twenty-one anonymous, naïve participants (45 women, median age = 30 years, an additional participant was recruited due to an error in counterbalancing; additional demographic information for this and later experiments is available in the OSF repository at https://osf.io/5vbzp/) were recruited through the website Prolific (www.prolific.co). To pass prescreening for this experiment, participants should have completed at least ten experiments previously and had a total approval rate of no less than 99%. Participants additionally could not have participated in any of the labs previous experiments using similar paradigms. The experiment was approved by the Ethical Review Committee of the Faculty of Behavioral and Movement Sciences of Vrije Universiteit Amsterdam, and all participants indicated their informed consent prior to the experiment. The experiment lasted approximately 35 min, and participants were compensated with £4.50. Participant data were excluded and replaced if their accuracy or average reaction time was 2.5 standard deviations (*SD*s) away from the group average accuracy. The data from two participants were replaced in this way due to low accuracies and two for slow reaction times. Trials with reaction times more than two standard deviations from each participant’s mean reaction time were additionally removed (<1% of all trials). Incorrect trials were also excluded from the final analysis (8% of all trials).

#### Design and procedure

All experimental code and stimuli are available online at (https://osf.io/5vbzp/). Participants accessed the experiment using their personal computers via the experiment hosting website JATOS (Lange et al., 2015) after recruitment on Prolific. The experiment was created using OpenSesame (Mathôt et al., 2012) with JavaScript. The experiment, which started with 24 practice trials, comprised five blocks: three for training and two for testing.

Our experiment design used a feature search task where participants searched for a set target among heterogeneous distractors (Bacon & Egeth, 1994; Stilwell & Gaspelin, 2021; Theeuwes, 2004). Each trial started with a jittered fixation dot (500–750 ms), followed by the appearance of a ring of six unique green shapes (square, diamond, rhombus, trapezoid, pentagon, hexagon), each with a grey line either tilted horizontally or vertically embedded within (three horizontals and three verticals on each trial). Participants were randomly assigned a target shape at the beginning of the experiment and had to report the orientation of the embedded line within this shape using the left and up arrow keys on their keyboard. Search arrays remained on-screen for 2,000 ms or until participants provided a response. Participants received feedback via the fixation dot (blinking green for correct or red for incorrect). Critically, unbeknownst to the participants, during practice and training, one of the five nontarget shapes appeared disproportionately often in one location (50% of trials, location counterbalanced across participants). The target appeared equally across all locations.

After three training blocks, participants received a new search target for the remaining two testing blocks. This target shape was always the shape that previously had a spatial regularity in the training phase. In the test phase, all shapes appeared with equal probability across all locations. Participants were informed before training that they would be given a new search target at some point in the experiment but were not told the shape this second target would be. Following testing, to assess awareness, participants were asked whether they noticed a regularity among the nontarget shapes and to identify its high-probability location. If a participant both indicated that they noticed a regularity and were able to correctly identify the shapes high-probability location, the participant was labelled as being aware.

### Results

We first investigated whether participants were significantly faster when the regular shape was at its high-probability location during training. Such a finding has been reported for salient, pop-out distractor singletons regardless of whether participants search for unique elements (the so-called singleton detection mode) or search for a specific target (feature search mode; Wang & Theeuwes, 2018b). In the training phase, if the same suppression effect was at play, then we would expect to see faster reaction times when the regular distractor appeared at its high-probability location, and slower reaction times when the target did. Yet, for the testing phase, experimental predictions are less clear. If participants are able to learn the nonsalient distractor’s regularity in training, when this distractor switched to being the target, this may either lead impaired performance at the previous high-probability location (because this location is still suppressed) or enhancement (because participants generally expect the shape to be there, with no regard to its role as a target or distractor).

**Fig. 2:**
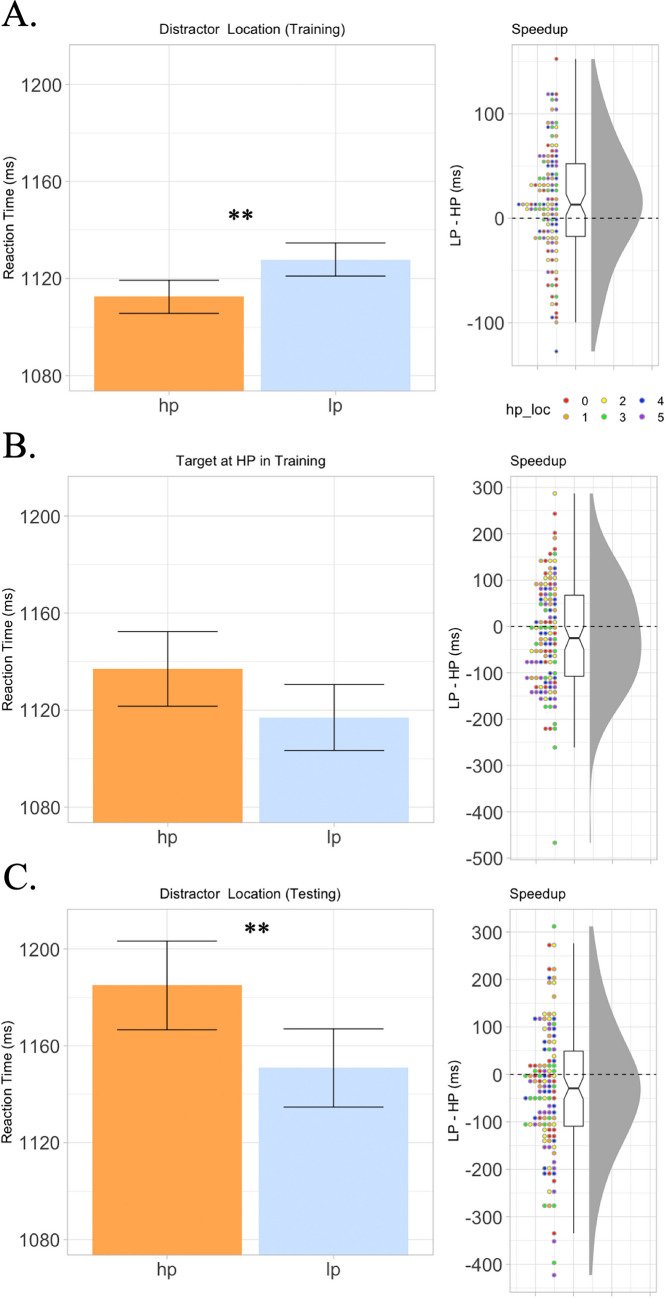
Stimuli and results of Experiment 1. **A**. Left: Reaction times when a nonsalient nontarget shape appeared at its high-probability location (hp) versus another low-probability location (lp). Error bars are adjusted for within-subject comparisons (Cousineau et al., 2021). Right: Individual participant speedup scores, calculated by subtracting each participant’s average reaction time on HP trials from their mean reaction time on LP trials. Dots indicate individual participant’s scores. Colors indicate different high-probability locations (hp_loc; legend on bottom left. 0 indicates top screen location, numbers count clockwise from there). Notches in boxplot indicate 95% confidence interval of mean. **B.** Participant means and speedup effect when targets are presented at the high-probability distractor location. Note that targets appeared at each of the six locations equally frequently. **C.** Reaction times and speedup effect when targets present at their previously high-probability location during the testing phase of the experiment. Note that target presentation rates were equalized in the testing phase of the experiment; any effect observed must be a carryover from the training phase of the experiment. **p < .01. (Color figure online)

Even though in the current experiment the critical distractor was not salient, there was still a reliable speedup effect when the nontarget shape was present at its high-probability location relative to its low-probability location, *t*(120) = 3.15, *p* = .002, *d* = 0.287 (Fig. 2A). When the target shape was presented at this high-probability location, there was a trend toward slower responses, but it was not statistically significant, *t*(120) = 1.89, *p* = .062 (Fig. 2B). Having established sensitivity to the nontarget shape's regularity during training, we examined if this learning affected selection during the test phase when participants had to find the shape that previously possessed a spatial regularity. A significant slowdown occurred when the new target shape was at its previously high-probability location, *t*(120) = 2.72, *p* = .008, *d* = 0.247 (Fig. 2C).^1^

Among 49 participants who reported noticing the nontarget shape’s regularity, only 10 correctly identified the high-probability location (chance level = 8). Including awareness as an additional factor did not yield significant interactions in the analyses of variance (all *F* values < 1), and results remained consistent when excluding these participants.

### Discussion

Despite the nontarget shapes being nonsalient and task irrelevant, participants nevertheless were sensitive to the embedded regularity.^2^ During training, they were faster when the regular nontarget shape was at its high-probability location. In addition, in the testing phase when this nontarget shape became the target, they were consistently slower when it appeared at its old high-probability location. This suggests that participants learned to avoid this location during training when it had the nontarget shape, and this bias persisted during testing, resulting in exaggerated negative shape priming at that location (DeSchepper & Treisman, 1996).

It has been proposed that VSL typically involves weight changes in the priority map, leading to proactive suppression or enhancement of certain locations based on their likelihood of containing a distractor or target (Duncan, van Moorselaar et al., 2023b; Ferrante et al., 2018; Huang et al., 2021, 2022). Per this explanation, it is possible that participants learned that a particular location was associated with a certain irrelevant shape and avoided orienting attention to this location. However, inconsistent with this explanation, during training, target processing at the high-probability distractor location was not reliably impaired (although a numerical trend in this direction should be noted). An alternative possibility is that rather than proactively suppressing this location, participants instead may have more quickly disengaged from stimuli in this location (Di Caro et al., 2019; Sauter et al., 2021; Wang et al., 2019). This disengagement would then appear closely linked to feature identity, where participants were better able to disengage from a certain distractor identity at a certain location in space.

While the results of Experiment 1 seem to offer a striking example of VSL without attention, several task features make it tenuous to claim that attention was never directed towards the nontarget shape with a regularity. Firstly, the search target and regular distractor shared the fundamental feature of color, meaning that the nontarget distractor may have still attracted some nonzero level of attentional processing (Forschack et al., 2017; Saenz et al., 2002; Störmer & Alvarez, 2014; Treue & Trujillo, 1999). Secondly, in feature search tasks, it is debated whether participants use parallel or serial search for target shapes in displays inducing the feature search mode (Leber & Egeth, 2006; Liesefeld et al., 2016; Liesefeld & Müller, 2019; Theeuwes, 2004, 2023; Wang & Theeuwes, 2020). If serial search is used, then participants would randomly scan across items in the scene until the target is found (Jonides & Yantis, 1988; Wolfe, 1994, 2021), a strategy that would lead to transient orienting of focused (top-down) attention towards the shapes with a spatial regularity. This incidental attentional orienting might then be sufficient for statistical learning to occur. Experiment 2 was designed to test this alternative hypothesis; during training, half of the shapes were colored red and the other half green. Previous research has shown that during serial search, participants can direct attention selectively to one target-matching color (Egeth et al., 1984; Kaptein et al., 1995; Lien et al., 2021; Nakayama & Silverman, 1986) ignoring colors of a nonmatching set. Critically, we made sure that the shape containing the regularity and the search target never had the same color, ensuring that selective attention would never be directed towards the subset of elements that contained the regularity during training (for a similar approach, see Jiang & Leung, 2005; Vadillo et al., 2020). This design allowed us to investigate whether the learning observed in Experiment 1 can be attributed to the occasional direction of attention towards the regularity due to the use of a serial search strategy.

## Experiment 2

### Method

#### Participants

Experiment 2 was preregistered following reasonable expectations derived from Experiment 1 (https://osf.io/xg6cn). A total of 121 new anonymous, naïve participants (median age = 32, 31 women) were again recruited through Prolific using the same screening criteria and run using the same online test hosting platform JATOS. Four participants were excluded and replaced in this experiment for low accuracies, and three for slow average reaction times using the same criteria as in Experiment 1 (2.5 *SD*s from group mean).

#### Design and procedure

**Fig. 3:**
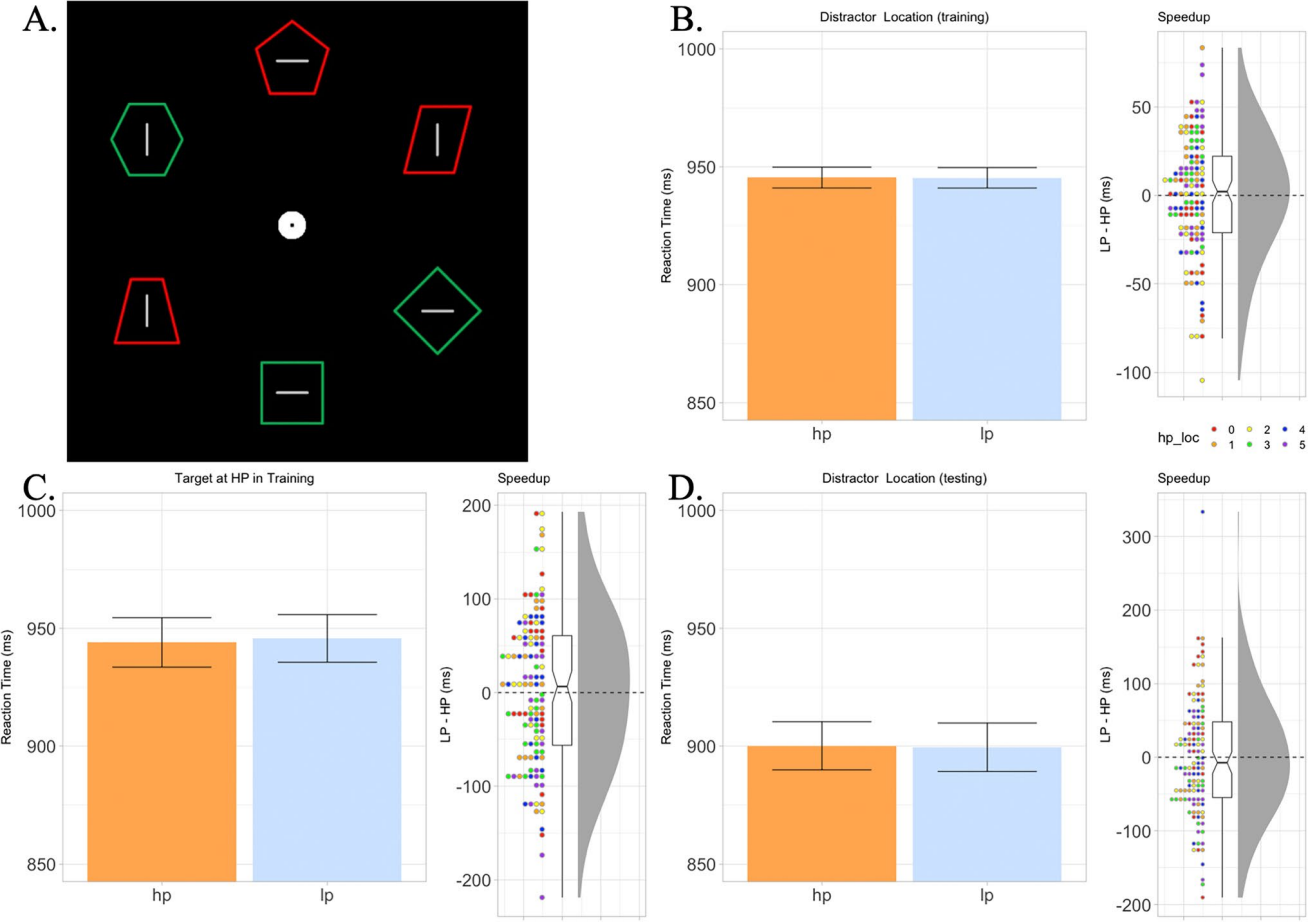
Stimuli and results of Experiment 2. **A**. Example of the two-color feature search task. The task in Experiment 2 was identical to Experiment 1, except that two shape colors were used and the search target in the training and testing phases always had a different color feature. **B**. Left: Mean reaction times when the nonsalient nontarget shape was at its high-probability location. Error bars are adjusted for within-subject comparisons (Cousineau et al., 2021). Right: Individual participant speedup scores, calculated by subtracting each participant’s average reaction time on HP trials from their mean reaction time on LP trials. Dots indicate individual participant scores; colors indicate different high-probability locations (legend on bottom right). Notches in boxplot indicate 95% confidence interval of mean. **C.** Participant means and speedup effect when targets are presented at the high-probability distractor location. **D.** Reaction times and speedup effect when targets present at their previously high-probability location during the testing phase of the experiment. (Color figure online)

Experiment 2 matched the procedure of Experiment 1, except for one key difference: At the beginning of the experiment, three shapes were randomly chosen to be presented in the color red, and three to be presented in the color green (Fig. 3A). These shapes remained the same color for the entire experiment. Similar to Experiment 1, the target during training was selected at random; however, it was additionally controlled such that the nontarget shape with a spatial regularity was a different color (e.g., if the target was red, the shape with a spatial regularity would be green). This meant that participants switched from searching from a target of one color to another color when moving from the training phase to the testing phase, where the shape with a regularity became the new search target. Each location was further controlled such that they contained a green shape on 50% of the trials and a red shape on 50% of the trials.

### Results

As in Experiment 1, incorrect trials were excluded (5% of total trials), and trials which were more than two standard deviations faster or slower than the individual participants mean reaction time were also excluded (<1% total trials). Counter to Experiment 1, there now was no evidence that participants were significantly faster when the nontarget shape appeared in its high-probability locations in this version of the experiment (*t* < 1; Fig. 3B) nor when targets appeared at this high-probability distractor location (*t* < 1; Fig. 3C). In line with these null findings, in the testing phase there was no evidence that participants were significantly slower when the new search target appeared at its previous high-probability location (*t* < 1; Fig. 3D).

To solidify our null findings, an additional Bayesian analysis was run for our three conditions of interest testing for evidence for a null distribution (BF_10_) using the default settings of the statistical analysis program JASP (Wagenmakers et al., 2018). Strong evidence for the null was found in all three of our conditions of interest (BF_01_ = 9.897; BF_01_ = 9.868, & BF_01_ = 9.643, respectively; analysis file can be viewed at https://osf.io/5vbzp/) providing significant evidence that reaction times did not vary based on the position of the regular shape during training or after it became the search target in the testing phase.

While a similar number of participants answered yes when asked if they noticed a regularity to one of the shape stimuli (39), only three participants were able to select the correct high-probability location. Again, their data did not significantly influence any of the previous analyses (all *F* values < 1).

### Discussion

While the results of Experiment 1 seemed to indicate that statistical learning during visual search is also observed when the item with a spatial regularity never captured attention in either a top-down or bottom-up way, in Experiment 2, where the nontarget shape was rendered in a task-irrelevant color, no signs of learning were observed. Together these findings demonstrate that while statistical learning of nonsalient shapes can occur when they are incidentally attended (because they share a task-relevant feature), such learning is absent when attention is allocated elsewhere. Thus, some degree of attention to the critical regularity is needed for VSL to occur.

A classic indicator of serial search is a monotonic increase in search times as the number of items in a search display increases (Theeuwes, 2004; Treisman & Sato, 1990; Wolfe, 1994). Consistent with this claim, the mean RT in Experiment 2 (~950 ms), where search could be limited to on average two items (between 1 and 3 items) was markedly reduced relative to Experiment 1 (~1,140 ms), where search had to discriminate on average 3.5 items (between 1 and 6 items). This monotonic decrease indicates that a serial search strategy was used to perform the task; a condition in which ignoring off-color distractors is highly efficient (Egeth et al., 1984; Kaptein et al., 1995; Theeuwes, 2023). Furthermore, VSL effects having been demonstrated in the past for searches as low as two items (van Moorselaar et al., 2020; van Moorselaar & Slagter, 2019), meaning there is no a priori reason that participants should not have been able to learn spatial regularities in a three-item experiment rather than a six-item experiment. Together with previous findings, it becomes clear that VSL can occur if regularities receive either top-down or bottom-up attention (or both), but crucially here it is shown that when stimuli receive neither, learning is not found.

While the current results show that statistical regularities regarding specific items are not learned when these items are filtered out by using attentional strategies, color subset selective search that we used here is just one method out of excluding a specific set of items. To expand on these results, in Experiment 3 we used exogenous cueing to direct attention away from the items that contained the regularity.

## Experiment 3

### Method

#### Participants

The preregistration for Experiment 3 can be viewed online (https://osf.io/kyd94). Unlike Experiments 1 and 2, Experiment 3 utilized a Bayesian stopping rule (Rouder, 2014) whereby results were monitored during data acquisition, and once a certain level of evidence (as measured by Bayes factors) was recorded, data collection was stopped. As per the preregistration, data collection continued until a Bayes factor (B_01_) of 6 was recorded for our critical analysis of interest (reaction times between HP and LP target trials in the testing phase). Using this rule, 85 participants’ data were collected (median age = 28, 41 women) using the same methods and prescreening criteria as in Experiments 1 and 2. In this dataset, four participants were excluded for low accuracies and two for slow reaction times using the same criteria as in Experiment 1 (2.5 *SD*s from group mean).

#### Design and procedure

**Fig. 4:**
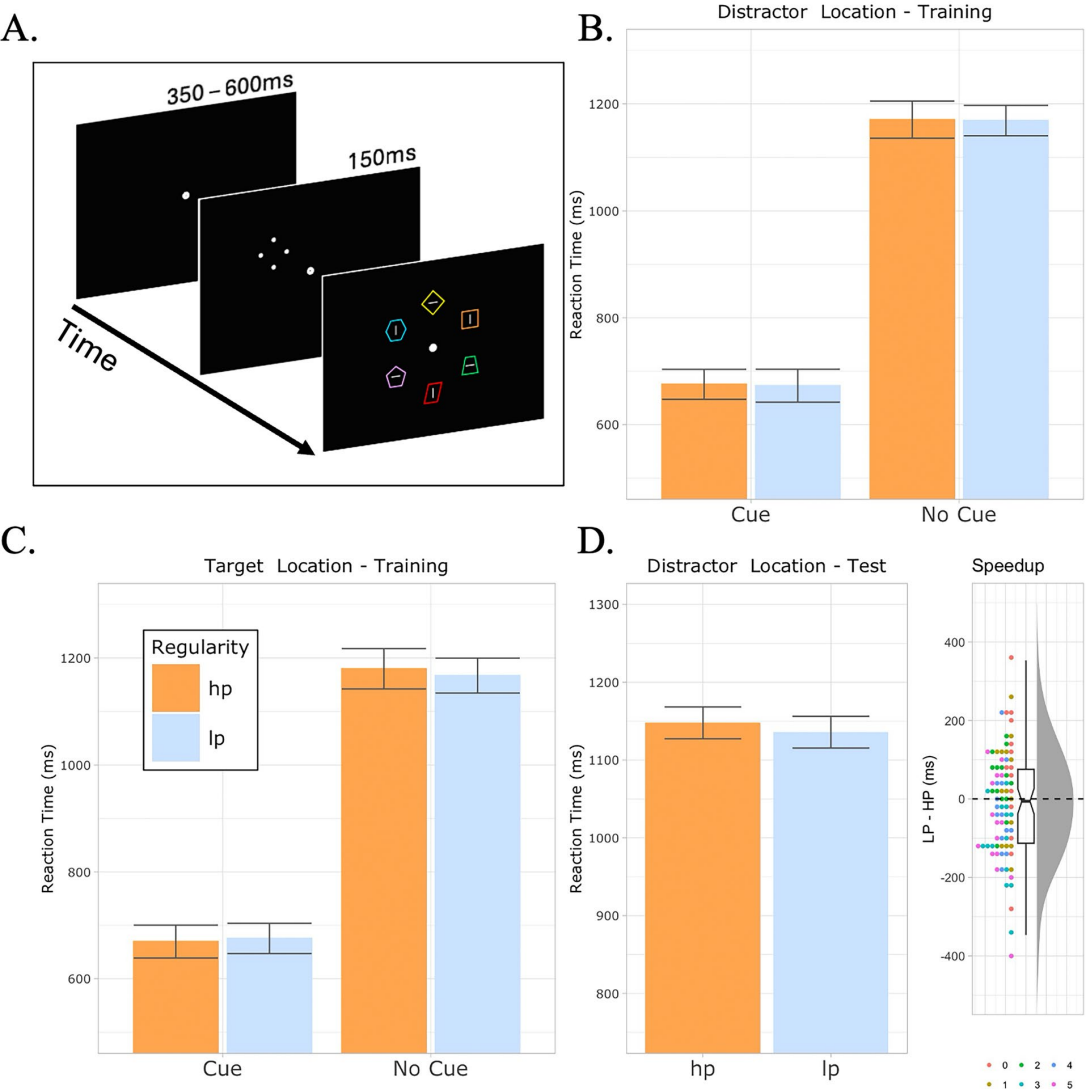
Stimuli and results of Experiment 3. **A.** Example of the experimental procedure including the pretrial cue. The task in Experiment 3 was identical to Experiment 1, except that during the training phase, immediately before search array onset, an exogenous cue would be presented for 150 ms. This cue was present on 75% of trials and had 100% validity. Importantly, on the 25% of the trials in which no cue was presented, trials were selected such that the target and distractors were equally present at all locations. In this way, on trials in which participants were compelled to carry out serial search, there was no spatial regularity to be learned. Search displays remained on screen until a response was provided or 2,000 ms expired. **B.** Mean reaction times during training when the nonsalient nontarget shape was at its high-probability (orange) or a low-probability (blue) location, separated between trials in which the cue was present or absent. Error bars are adjusted for within-subject comparisons (Cousineau et al., 2021). **C.** Participant mean reaction time when the search target was presented at the high-probability distractor location, again separated between cue and no-cue trials. **D.** Left: Reaction times during the testing phase when the target happens to be presented at the location that was a high-probability location during the training phase. Right: Individual participant speedup scores, calculated by subtracting each participant’s average reaction time on HP trials from their mean reaction time on LP trials. Dots indicate individual participant scores; colors indicate each participant’s high-probability location condition (legend on bottom right; 0 indicated top of screen and progresses clockwise). (Color figure online)

Experiment 3 exactly mirrored the design of Experiment 1 except for two new features. First, during training, immediately before trial onset, an exogenous cue could appear indicating with 100% validity where the search target was about to be displayed (Fig. 4A). This exogenous cue was presented on 75% of trials. Importantly, on no-cue trials, a subset of trials was selected such that the target and regular distractor appeared at each location the same number of times, implying that the regularity was only present in those trials in which the cue was presented. The cue was only shown during the training phase of the experiment, meaning all trials in the testing phase were no-cue trials. Additionally, on each trial, shapes were rendered in a random color, indicating that unlike in Experiments 1 and 2, the search template was color neutral (for a similar design, see Kim et al., 2023, Experiment 1).

### Results

As in previous experiments, incorrect trials were excluded (5% of total trials) and trials which were more than two standard deviations faster or slower than the individual participants mean reaction time were also excluded (<1% total trials). Given an optional stopping rule was utilized, the reporting of frequentist statistics would be inappropriate (Sanborn & Hills, 2014) and so only Bayesian analyses are reported here. For the training phase analysis, a Bayesian repeated-measures analysis of variance (rANOVA) taking cue present/absent and high/low probability distractor conditions as factors and reaction time as the dependent measure selected the best performing model to be one with only a main effect of cue presence. This cue-only model strongly outperformed the second-best model: one with cue and distractor location effects but no interaction (BF_01_ = 6.7; Fig. 4B) indicating that participants effectively used the cue to improve their reaction times but were unable to utilize the distractor information in the same way as was previously observed in Experiment 1. A similar pattern was found when analyzing the target location during training, with a cue-only model outperforming a second-best cue + distractor location model (BF_01_ = 6.3; Fig. 4C). The primary analysis of interest in this experiment was how reaction times would differ during the testing phase where the target appeared at its previously high-probability distractor location. As per the preregistration, a Bayesian *t* test strongly favored the null hypothesis (BF_01_ = 6.05, Fig. 4D; for analysis file of all the above analyses, see https://osf.io/depfg). Similar to Experiment 2, the current results show no evidence of learning when attention is effectively shielded from a visual regularity.

Once again, only a small subset of the participants that reported noticing the regularity (34) were actually able to report where the previous high-probability location was (6). Again, their data did not significantly influence the previous analyses (all *F* values < 1).

## General discussion

The present study was designed to investigate whether statistical learning would continue to adjust attentional priorities when the embedded regularity across visual searches would receive neither top-down nor bottom-up attention. For this purpose, a nonsalient shape within a heterogeneous search display appeared with a higher probability at a given location in the search display. In Experiment 1 it was found that search was facilitated when the regular item appeared at its high-probability location, seemingly suggesting learning with neither top-down nor bottom-up attention. However, the experimental design left open the possibility that the regular distractor was occasionally transiently attended due to its shared low-level features (i.e., color) with the search target. To control for this possibility, in Experiment 2 the colors of the search items were manipulated such that during training the nonsalient distractor with a spatial regularity was a different color than the current search target. After having eliminated the possibility that attention would randomly be oriented towards the regular distractor, there was no longer any hint of learning, indicating that statistical learning requires at least some attention to the critical regularity. Following up on this result, Experiment 3 replicated the results of Experiment 2 but using a different attentional guidance technique, thereby demonstrating that the null effect was not specific to color-subset search. Specifically, exogenous cuing was used to effectively direct participants’ attention away from the distractor regularity during training. Importantly, while previous work has robustly demonstrated that top-down attention can directly affect the expression of statistical learning effects (Dolci et al., 2023; Richter & de Lange, 2019), no studies have yet definitively shown whether attention is needed for the learning of the regularities themselves. Using this alternate attentional manipulation, the same null effect was observed as in Experiment 2, demonstrating the generality of the finding.

Several influential theories on the mechanisms underlying statistical learning have theorized that attention is a necessary component for learning to occur (Anderson et al., 2021; Theeuwes et al., 2022). This question poses theoretical consequences for the larger concept of statistical learning in distinguishing it from low level phenomena such as habituation and neural adaptation, which are essentially attention free (Larsson & Smith, 2012; Maffei et al., 1973; Vautin & Berkley, 1977). The current results are consistent with these theories, demonstrating that attention is indeed a crucial ingredient for statistical learning. While the current findings convincingly demonstrate that VSL is no longer observed when the regularity receives neither top-down nor bottom-up attention, the fact that learning was observed when only controlling for bottom-up attention highlights the visual system’s remarkable sensitivity to regularities within the environment. Whereas previous studies demonstrating statistical distractor learning already demonstrated that VSL is more ubiquitous than previously thought (see Theeuwes et al., 2022, for a review), the current findings take this one step further: showing that even a minimally, transiently attended stimulus can entrain some form of learning. Furthermore, the benefits observed in the training phase reversed in the testing phase, slowing reaction times when the previously regular item became the search target. This effect did not generalize to the training target, indicating that the test target shape itself was processed worse at its high-probability location (though a trend towards training target suppression was also found). These results agree nicely with a series of experiments by Failing, Feldmann-Wüstefeld et al. (2019a), Failing, Wang et al. (2019b) where it was shown that both feature and spatial distractor regularities could be simultaneously learned and interacted, where certain feature-defined distractors were better suppressed at their high-probability locations. The current results support such a feature–space binding in statistical learning where rather than improving search efficiency by blindly suppressing one region of space, the processing of distracting features themselves were affected at suppressed regions. Additionally, the current study is another in a growing collection of studies showing feature-based statistical learning of either distractor (Kim et al., 2023; Ogden et al., 2023; Stilwell et al., 2019; Won & Geng, 2020) or target features (Conn et al., 2020; Sha et al., 2017; Wang et al., 2023) in visual search tasks.

Together, these experiments elucidate the interaction of attention and statistical learning, which has been a question of interest since early work by Saffran and colleague’s demonstrated the seemingly automatic ability of infants to learn grammatical regularities (Saffran et al., 1996, 1997). Since then, a wealth of evidence has provided conflicting proof for both camps, with compelling demonstrations of the need for attention for visual learning to occur (Jiang & Chun, 2001; Turk-Browne et al., 2005; Vadillo et al., 2020) or for the opposite—the presence of VSL without attention (Duncan & Theeuwes, 2020; Jiang & Leung, 2005; Musz et al., 2015; Turatto et al., 2018). Several works in particular warrant highlighting: The seminal work of Turk-Browne et al. (2005) suggested that participants could only learn regularities related to task-relevant stimuli using a triplet learning paradigm. Their results were challenged, however, by subsequent replications which showed that if a larger sample was collected, learning could also be observed for task-irrelevant items (Campbell et al., 2012; Musz et al., 2015). Importantly, this learning is likely due to attention being drawn towards the items in an exogenous way as they were presented alone on the screen, agreeing with recent results that suggest bottom-up attention is sufficient for learning to occur (Duncan & Theeuwes, 2020; Turatto et al., 2018; Won & Geng, 2020). Baker et al. (2004) cleverly demonstrated the role of attention in shape-pair learning. Connecting shapes with a bar, thus evoking object-based attention, led to shape contingency learning, while no pair learning occurred when shapes were disconnected. However, post hoc testing revealed participants’ explicit knowledge of shape contingencies, presenting a challenge to generalizing their results to other VSL tasks which are often seen as implicit (e.g., learned distractor suppression). Furthermore, it is known that bottom-up-driven learning effect sizes are reliably smaller than when top-down attention is used (Duncan & Theeuwes, 2020; Jiang & Chun, 2001; Musz et al., 2015), and given their already barely detectable reported effect sizes, it is unclear if evidence of learning of the unattended regularities would be found in a larger sample (Brysbaert, 2019). Finally, in contextual cuing the work of Jiang and Chun (2001) showed that participants could use the global configuration of a display when it predicted target location only when these repeated contexts were presented in a task-relevant color. These results, however, were later challenged by the first author of the original paper in a new design demonstrating that participants could in fact learn this ignored spatial context as well (Jiang & Leung, 2005), thereby supporting the opposite attention-independent perspective on statistical learning. This conflict as well as difficulties in replicating these results (Vadillo et al., 2020), and recent questions about what is learned in contextual cuing (Meyen et al., 2023; Seitz et al., 2023) make the results of these studies difficult to interpret.

In the current study, by carefully controlling the influence of both top-down and bottom-up attention in a large sample size sensitive to small effects, the concerns which have impacted previous studies were addressed. The results of these experiments ultimately support the assertion that VSL depends on attention to occur. It is important to note that the current results demonstrate attention dependent learning on one form of VSL, but caution should be taken in generalizing these findings to other, non-VSL modalities as there is considerable debate whether the diverse family of statistical learning paradigms all originate from one domain-general statistical learning mechanism (Arciuli, 2017; Conway, 2020; Frost et al., 2015). Furthermore, work in nonvisual domains have been highly mixed in regard to the influence of attention on statistical learning, with both audio statistical learning (Batterink & Paller, 2019; Fernandes et al., 2010; Saffran et al., 1997; Toro et al., 2005) and haptic statistical learning (Frensch et al., 1998; Horváth et al., 2020; Janacsek & Nemeth, 2013; Jiménez & Mendez, 1999) showing mixed results in often underpowered studies (Brysbaert, 2019). Additionally, there is debate whether VSL paradigms also differ in fundamental mechanisms within the visual domain (Ferrante et al., 2018, 2023), leaving open whether the same attention-dependent mechanisms observed here would be in place for, for instance, target contingency learning. Thus, there remains clear motivation to further study the role of attention in other statistical learning paradigms. In sum, the current findings highlight the profound interplay between attention and learning, and support the broader assertion that attention is a fundamental filter through which information must pass to be learned.

## Data Availability

All anonymized experiment data, experiment script, analysis code, and preregistrations are available online (https://osf.io/5vbzp/).

## Footnotes

1 For the sake of consistency with the statistical approach used in Experiments 2 & 3, the Bayes factors (BF_10_) for the above reported effects were 7.881, 0.455, and 3.452 for the distractor effect in training (Fig. 1A), target effect in training (Fig. 1B), and target effect in testing (Fig. 1C), respectively.

2 Note that these results are an exact replication of an unregistered pilot experiment included in the OSF archive.

## Code availability

This is stated in the open practice statement.
